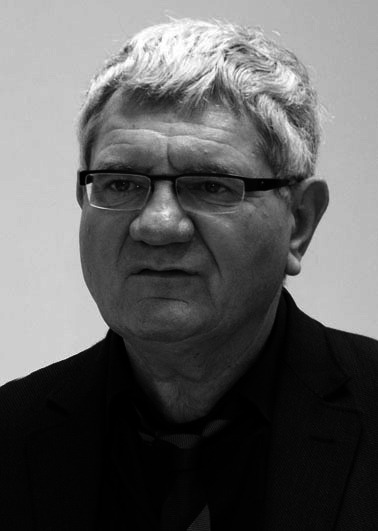# Chemical Batteries with CO_2_

**DOI:** 10.1002/anie.202007397

**Published:** 2021-12-16

**Authors:** Robert Schlögl

**Affiliations:** ^1^ Max-Planck-Institut für Chemische Energiekonversion Stiftstrasse 34–36 45470 Mülheim an der Ruhr Germany; ^2^ Fritz-Haber-Institut der Max-Planck-Gesellschaft Faradayweg 4–6 14195 Berlin Germany

**Keywords:** chemical batteries, energy conversion, methanol, synthetic fuels

## Abstract

Efforts to obtain raw materials from CO_2_ by catalytic reduction as a means of combating greenhouse gas emissions are pushing the boundaries of the chemical industry. The dimensions of modern energy regimes, on the one hand, and the necessary transport and trade of globally produced renewable energy, on the other, will require the use of chemical batteries in conjunction with the local production of renewable electricity. The synthesis of methanol is an important option for chemical batteries and will, for that reason, be described here in detail. It is also shown that the necessary, robust, and fundamental understanding of processes and the material science of catalysts for the hydrogenation of CO_2_ does not yet exist.

## Graphical abstract

Sustainable energy systems cannot exist without the storage of volatile renewable electricity. Whereas electrical batteries can be used for small amounts of energy, chemical batteries are required for large amounts of energy. The hydrogenation of CO_2_ is one promising option for chemical batteries. The intricate material science of Cu catalysts to control the selectivity of this reaction is discussed in detail in this Review.

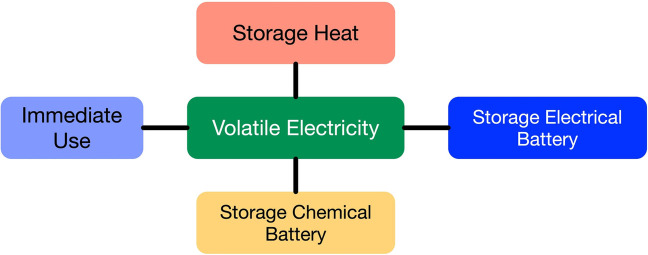

## 1 Introduction

Current Research considers the hydrogenation of CO_2_ from three perspectives. There are few chemical reactions that concern so many people outside of chemistry as does the future processing of CO_2_. In reference to these efforts, we use the abbreviation “CCU” (Carbon Capture and Use). The use of CO_2_ is first considered in the context of sustainable energy regimes. Here, the focus is directly on the storage and transport of sustainably produced hydrogen in the form of “synthetic fuels.” The notion should not be entertained here that hydrogen is a means of removing CO_2_ from the atmosphere. Rather, CO_2_ is a component of a circular economy for renewable energy. This concept will be expressed more thoroughly in the introduction to chemical batteries. As with electrical batteries, there is a chemical accumulator (or rechargeable battery) that can be repeatedly discharged and charged in a closed cycle. There is also a type of electrical battery that cannot be recharged. This device corresponds to the single‐use binding of CO_2_ to renewable hydrogen in synthetic fuels, which is referred to in the literature as “linear CCU” in contrast to the use of repeatedly (through biological or technical processes) collected CO_2_ that leads to “circular CCU”.

The concept of the battery is not only a quaint formulation, but also an attempt to draw the attention of energy regime regulators to the fact that CO_2_ prices and taxes must be divided into three categories if a successful incentive is to be achieved. These categories are: no utilization (max price), linear use (discounted price), and circular use (no price). For each case, different definitions and rules apply.

A significant aspect—and one that is often treated in a cursory fashion—is the use of CO_2_ as a raw material in the chemical industry. Here, many synthetic routes and concepts for novel production chains exist. They are of importance for today's chemical industry and will continue to be so in the future.

A large part of this Review is devoted to understanding the reaction of CO_2_ with hydrogen, whereby copper is used as a thermochemical or electrochemical catalyst. Beginning with a network of reactions, it will be shown that the term “copper metal” is too simple a term to describe the active component in the catalyst if the goal is to understand the different effects of nominally the same copper catalyst in the reaction network.

**Figure 1:**
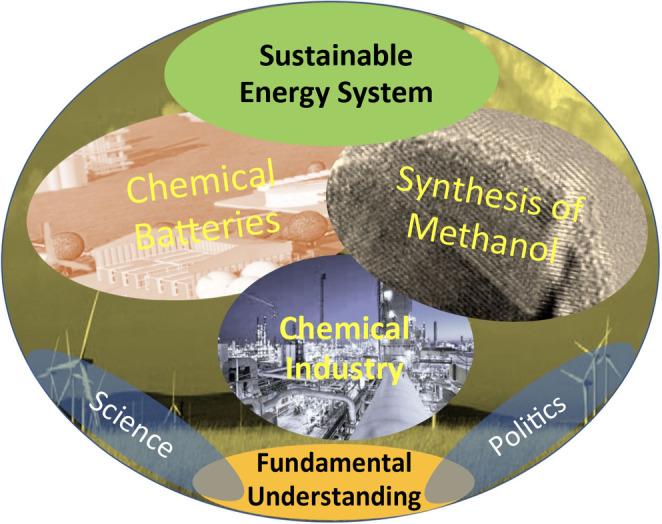
The topics of the current Review. The selected subjects are, taken together, important for a successful energy transition. This transition can only be realized collectively by the stakeholders from science and decision‐making standing behind the elements shown in Figure 1. It would be highly desirable if all actions were based upon the fundamental insights existing today.

Figure 1 shows a schematic “work program.” It is geared toward different target audiences of chemists and policy makers active in the energy sector. This facilitation of information must take place while recognizing that the challenge of restructuring the energy regime can only be successful as a communal effort and with a solid grasp of the tasks and possibilities. Researchers may see that the description of the underlying processes of their phenomenological research is a motivating factor for their work. Policy makers should come to understand the high accuracy with which we can already describe these central processes—but also be aware that some knowledge and transformations into robust technologies are still lacking. In this way, both groups can come face‐to‐face with the complexity of such an undertaking as energy transition.

## 2 Chemical Batteries

Batteries are devices that store energy. The name comes from military speech and refers to the strengthening of an effect or action by organizing single units into groups. In electrochemical batteries,^[1]^ a series of redox reactions are carried out within electrically connected cells. Free electrons produced during the course of the reactions can then perform work in an external electric circuit. The necessary energy for this process is stored in the electrodes of the battery. The interconversion between chemical and electrical energy takes place with only small losses because the oxidation state of the storage medium—the ion—can easily be changed. For some combinations of redox reactions, the storage process is reversible and the application of external electrical work can reverse the process with, again, only small losses.^[2]^ In these cases, one speaks of accumulators or rechargeable batteries.

One disadvantage of both rechargeable and non‐rechargeable batteries is that the energy is stored in the device—specifically in the solid electrodes. The alternative redox‐flow battery^[3]^ avoids this disadvantage by including liquids as the storage medium for the ions, which can be held externally from the charge conversion device. This form of accumulator is, however, not yet fully developed and cannot store large quantities of energy. If the goal is to store electrical energy in quantities on the order of magnitude of the demand of entire countries, then chemical batteries are essential to make them globally transportable, for example, or to de‐fossilize applications and processes requiring high energy densities. Such batteries consist of molecules containing energy stored in chemical bonds. For example, hydrogen, methane, or other alkanes, are often used for this purpose and are generally well‐known today as fuels. In chemical batteries, the processes of storing and recovering the energy is separated from the storage form itself. For this reason, there is no limitation on the amount of energy that can be stored or the duration for which it can be stored. This advantage of almost unlimited storage capacity is, however, accompanied by the disadvantage that further reaction partners are required for the energy storage and recovery processes themselves. The following reactions illustrate the difference between electrochemical batteries and chemical batteries with free electrons [Eq. (1,2)] and chemical bonds [Eq. (4,5)], respectively, as their storage medium.

Electrochemical battery:

Storage:

(1) $$\mathrm{Li}{}^{+}+e{}^{-}\to \mathrm{Li}$$

(1a) $$\mathrm{LiM}{}_{x}O{}_{y}\to 1-z\;\mathrm{Li}+\mathrm{Li}{}_{z}M{}_{x}O{}_{y}$$

Recovery:

(2) $$\mathrm{Li}\to \mathrm{Li}{}^{+}+e{}^{-}$$

Equations (1) and (2) are simplified to illustrate the principle of storage using ions. Equation (1) implies that, in real batteries and accumulators, complex reactions^[4]^ take place between the storage ion and the electrode material. For a chemical battery, a further process [Eq. (3)] is necessary in addition to the reactions for storage [Eq. (4)] and recovery [Eq. (5)].

Chemical battery:

Primary conversion:

(3) $$2\;H{}_{2}O\to 2\;H{}_{2}+O{}_{2}$$

Storage:

(4) $$\mathrm{CO}{}_{2}+4\;H{}_{2}\to \mathrm{CH}{}_{4}+2\;H{}_{2}O$$

Recovery:

(5) $$\mathrm{CH}{}_{4}+2\;O{}_{2}\to \mathrm{CO}{}_{2}+2\;H{}_{2}O$$

Chemical batteries require a circular economy of storage molecules to enable a constant supply of energy; these molecules are a hallmark of a sustainable energy regime. Water, oxygen, and nitrogen molecules are present in such large quantities on Earth that no closed cycles are necessary. The purpose of the present Review is, however, to consider the suitability of CO_2_ as the storage molecule. For this material, a closed cycle is certainly necessary because of its multiple other functions in the atmosphere (greenhouse gas). The industrial revolution and all the resulting developments have depended on the high energy density of fuels and the resulting large amount of CO_2_ released through the use of these fuels. Concepts for carbon cycles were first suggested by Asinger and Olah.^[5]^

At a first glance, the lengthy process chain makes it appear that chemical batteries should perhaps be limited to hydrogen. The concept of a hydrogen economy^[6]^ is based on this perception. However, the unfavorable storage characteristics of hydrogen (low density, high energy requirements for liquification) in addition to technical hurdles in the process chain of hydrogen production, transport, and storage as well as conversion back into electricity by fuel cell technologies are, taken together, very demanding. The current expectation^[7]^ is, therefore, that a carbon‐based circular economy will run parallel to a hydrogen economy and will always have a particular role to play (materials, aircraft). Against the backdrop of the urgency for defossilizing energy regimes, the efficiency losses in a carbon‐based circular economy—which are largely understood technologically and can be well‐described economically—are acceptable when compared to the challenges of a hydrogen economy. Furthermore, the possibility of a shrewd combination of reactions (3) and (4) is evident. In this case, the losses associated with creating an H−H bond may be avoided because the bond is broken in the subsequent process. The price of bypassing this step requires the challenge of carrying out the liquid/gas (water/CO_2_) reaction in an electrochemical reactor,^[8]^ in which both phases react with one another at the electrode. The gas‐diffusion electrode offers a viable possibility. However, this is significantly more complex (at large scale) than the combination of conventional electrolysis with a solid–gas reactor.

There will be many application scenarios for a circular economy of energy carriers and will include centralized as well as decentralized solutions.^[9]^ Therefore, it is advisable to investigate and develop all viable pathways for achieving technical maturity. In this way, the user of a technology will have a range of possibilities from which the best systemic solution can be chosen. It is, therefore, necessary to exploit all possible gains in efficiency^[10]^ in chemical batteries. For these improvements to take place, a science‐based and robust understanding of the fundamentals of the materials and processes is a prerequisite.

## 3 CO_2_ as a Raw Material with Value

The chemistry of CO_2_ has long been the subject of research.^[11]^ The motivation for these investigations has been triggered by the wide availability of the reactant^[12]^ as well as the desire for the construction of a circular economy^[13]^ based on carbon. For others, however, the thought of a chemical exploitation of CO_2_ is an atrocity or even a “thermodynamic crime”.^[14]^ Their arguments contend that the “love for the conversion of CO_2_”^[13a]^ is a waste of energy because CO_2_ lies so far down the energy scale. Those opposed to the thought of a CO_2_ cycle suggest deposition and confinement of the material as the only scalable means of combatting the greenhouse effect.^[15]^ Nature itself exhibits many carbon cycles and exploits these opportunities to store energy in different ways.^[16]^ CO_2_ plays an important role in these cases. For this reason alone, it is advisable to thoroughly study the chemistry of this molecule and its applications as a chemical battery.

To assess the thermodynamic arguments, several standard enthalpies of formation will be considered. In no way do such considerations substitute for a complete analysis of chemical energy storage processes. However, such analyses contain so many process‐specific values that the results lead to a few general conclusions. Detailed examinations of this kind can be found in the literature.[^11d^, ^17^] The standard enthalpy of formation of CO_2_ is, at a value of −393 kJ mol^−1^, 318 kJ mol^−1^ more negative than the value of methane, which will be used here as a general, or standard, energy carrier. If CO_2_ is allowed to react with a typical base such as Ca(OH)_2_ to form the corresponding carbonate, 1207−393=814 kJ mol^−1^ are gained—a significant value for a supposedly low‐energy^[18]^ molecule. This value also illustrates the care required for underground storage of large quantities of CO_2_. Although mineralization processes chemically bind the CO_2_, they may also have a large number of effects on the bedrock.

Table 1 provides some orientation for the energetic relationships during “charging” and “discharging” of the chemical battery CO_2_. It should be remembered here that the initial energy investment required for the synthesis of one mole of storage molecules (charging) is accompanied by a release of enthalpy during the reaction. The difference in these values is equal to the enthalpy which remains stored in the system. The energy source for the charging step is hydrogen, which must be obtained from sources and processes which release no CO_2_.

**Table 1:** Selected thermodynamic data for reduction products of CO_2_.

Storage molecule	Energy investment for synthesis from CO_2_ [kJ mol^−1^]	Enthalpy released [kJ mol^−1^]	Stored enthalpy [kJ mol^−1^]	Primary loss [%]
methane	1521	638	883	42
methanol	1239	521	718	42
ethanol	2478	1123	1355	45
butane	4155	1275	2880	31

Table 1 also gives an impression of the “value” of selected molecules produced in the storage and transport of renewable energy. The rule of thumb for the storage of renewable electricity in organic storage media is that approximately half the energy is lost. This value represents a conservative lower limit and can be markedly improved by an astute selection of storage material and processing techniques (for example, utilization of the enthalpy released during the reaction). For the energy investment listed in Table 1, the storage molecules can be produced with intermittent renewable energy. They can be stored, transported, and converted using existing infrastructure and can, therefore, be considered in their functionality to be “green oil and green gas”.^[19]^

Although the value of CO_2_ hydrogenation is still being debated, the atmospheric concentration of CO_2_ has climbed to 408 ppm and is growing at 2.17 ppm each year.^[20]^ The question must be asked, what chemistry can do to reverse this trend, or at least stop it. Many answers have emerged^[21]^ and can be found in a diverse array of activities reported in the literature. An analysis of these answers^[22]^ must display the urgency of the task at hand as well as the complementary quantitative and temporal scalability of the chemical options. It should be clearly understood that the exploitation of CO_2_ as a raw material in the chemical industry, often viewed as a source of motivation in the literature, offers little in the way of greenhouse gas reduction when compared to the use of CO_2_ as an energy carrier. In Germany, 834 PJ equivalents of fossil resources were consumed by the chemical industry in 2018. This usage corresponded to 6.3 % of Germany's primary fossil energy requirement of 13 106 PJ. If the supply of raw materials for the chemical industry were shifted to CO_2_, a considerable increase in renewable electrical energy would be necessary to chemically reduce it. On average, three molecules of water would be needed to split one molecule of CO_2_, in addition to the energy required for the conversion processes themselves. The DECHEMA study “Roadmap Chemie 2050^[23]^” estimates an energy investment of 550 TWh for the chemical reduction of CO_2_ to hydrocarbon feedstock, which corresponds approximately to Germany's entire electricity consumption.

The products of the chemical industry then should be manufactured from CO_2_ if this results in simplified synthesis processes, or if waste and CO_2_ emissions can be reduced. This change is only sensible if the necessary energy (and hydrogen) are supplied solely^[11d]^ by renewable sources. Before this happens, chemical research should be tasked with finding processes and catalysts that reduce CO_2_ emission on a per‐product basis. Chemistry can also establish new, less‐intensive CO_2_ pathways for the supply of essential compounds. This is, in fact, a classic area of research in chemistry[^13a^, ^24^] and has continuously been a motivating factor in the search for new catalysts. At the same time, however, the main motivation has always been supplied by the minimization of raw material precursors and the avoidance of waste with the corresponding monetary savings. Today, chemistry's colossal task is to defossilize the resources that serve as the basis for the chemical industry.

Many products of the chemical industry are burned at the end of their lifecycle, or after recycling, whereby the CO_2_ still contained in them is set free. If these practices were made to complete a cyclic process that used the burned waste as a source of CO_2_, the cycle would remain closed. If control over the chemical products is lost, for example through landfilling, the integrity of the closed cycle cannot be guaranteed and the reduction of CO_2_ emissions^[25]^ is limited to 50 %. This maximum value corresponds to the reduction of fossil raw materials for the synthesis of chemical products and can only be reached^[25]^ if all necessary energy supplies are drawn from sources emitting no CO_2_.

Chemical research can also improve existing processes in the energy industry (coal, oil, gas). This relationship is old^[24b]^ and has contributed to the current high capacity and productivity of this industry. One fast and effective way to reduce CO_2_ emissions in this area would be the application of hydrogen, which is used in large quantities in the petrochemical industry, from fossil‐free sources (electrolysis, methane cracking^[27]^) and not from the more economical steam reforming of methane. Another option is to replace fossil energy carriers used for heat generation by electricity from renewable sources (or green hydrogen). Here, collaborative research between chemical engineering and material science is needed to find the best way for electrical energy to be introduced in chemical processes.

A central role for chemistry will be the storage and transport of renewable energy, which is initially produced as electricity, so that this energy can become a globally available traded commodity.^[28]^ This operation will require “green oil and green gas^[30]^” and depend to a significant extent on chemical batteries. Methane, methanol,^[30]^ LOHC,^[31]^ and ammonia^[32]^ are currently crucial substances for this purpose. The task of replacing fossil energy carriers will be difficult without the chemical reduction of CO_2_. It is for this reason that the chemically simple, but urgently needed products arising from the reduction of CO_2_ to “solar fuels” are the most valuable products^[33]^ in the defossilization of the energy industry despite their low specific economic added value when compared to chemically complex molecules from the chemical industry.

Finally, the direct replication of nature and the energy storage cycle (also based on the reduction of CO_2_ following photochemical water splitting^[34]^) could be one of chemistry's central contributions to the energy supply of the future. The natural photosynthesis of hydrocarbons occurs without the conversion of light into free electrons and the subsequent storage of the energy in chemical bonds. However, the process requires an extremely complex series of reactions which, at this time, cannot be imitated by technology. “Artificial photosynthesis” has been, and is still currently, the subject of intensive research.[^16b^, ^35^] However, it will not be discussed further here because the results of the research[^30^, ^36^] have not yet contributed significantly to the supply of chemical products at the focus of this text.

This author is of the opinion that all these possibilities are important and should continue to be pursued in chemical research. Evidently, this is currently the case and the results will provide a portfolio of options in the future. The urgency to act immediately to convert and store quantities of energy on the scale of today's oil and gas industry means that priority must now be given to the processes and materials needed for the restructuring of the present non‐sustainable energy systems, even if they are not optimal in terms of process efficiency. This perspective applies equally to process fundamentals as well as the mechanistic understanding and the identification of optimal functional materials.

Central questions surrounding the incorporation of CO_2_ reduction into energy regimes involve the production and purification^[37]^ of CO_2_ (catalyst poisons), running facilities on sources of intermittent energy^[38]^ (dynamic process management), and the question of facility size and complexity[^9^, ^39^] (centralized versus decentralized). Diverse scientific questions resulting from this situation have received only secondary priority,^[40]^ in part because they require experimental capabilities beyond the reach of typical academic research groups.

The processes of photosynthesis, biomineralization, and also technical mineralization^[41]^ of building materials show clearly that CO_2_ is a reactive molecule. The carbon contained in these materials may not be able to make a transition to a higher oxidation state, but the molecule is still reactive in many ways. It can, for example, generate different forms of organic and inorganic carbonates.^[42]^ CO_2_ can form adsorbates with metal surfaces, which have already been investigated for some time.^[43]^ The adsorbates then form the basis for the heterogeneous catalytic reduction of CO_2_ with hydrogen.

## 4 Valuable Reactions

The question considered here will be which reactions lead to valuable products through the reduction of CO_2_. To provide an answer, an overview of the current literature will first be provided and discussed. Along the way, the meaning of the word “valuable” should be kept in mind. The view is widespread^[13a]^ that every molecule can be considered valuable when the oxidation state of carbon is less than it is in CO_2_. Inorganic carbonates,^[44]^ such as molecules^[45]^ in which CO_2_ plays the role of a building block during synthesis, are also considered valuable. The view is also held that CO_2_ could be an important raw material^[24b]^ for the chemical industry when the current stock of oil and gas either become too expensive or are no longer viable due to defossilization. This motivation has led to the development of novel reactions which form complex microstructures and polymers with the CO_2_ building block. The molecular chemistry of CO_2_ has already been reviewed many times[^11a^, ^11b^, ^13^, ^45^, ^46^] and is treated only peripherally here. Interface catalysis[^21^, ^22^, ^28^, ^47^] of the CO_2_ reduction can be described in a similar way. The current Review uses the insight provided by these studies and makes an attempt at a critical analysis. This goal seems justified in light of the fact that the long history of research on the reduction of CO_2_ has led to a constriction of research foci and makes a satisfactory overview difficult. The formation of methanol will be considered here as an example of this situation.

A carbon cycle^[48]^ for the transport of renewable energy would certainly be the largest application of the hydrogenation of CO_2_. The size of today's oil and gas industry provides an impression of the required dimensions. One application of CO_2_ hydrogenation can be found in the production of synthetic fuels.[^21^, ^49^] At this time, although the opinion slowly abates that mobility should be completely electrified, it is especially important to intensively investigate the molecular structures which are in fact promising for use as fuels. Finding optimal pathways for their synthesis is also part of this search. The most valuable aspect of the chemical reduction of CO_2_ is not only its economical nature, but also pertains to the defossilization of the energy regime. It is noted here that this viewpoint is not shared by all[^25^, ^51^]—especially not by those who point to efficiency arguments about the conversion chain. Furthermore, the “leakage” of synthetic fuels in a cycle using CO_2_ as the energy carrier has also been criticized^[15]^ on the grounds that mobile sources burning carbon‐containing fuels emit CO_2_ and that the origin of the CO_2_ (from the air or biomass (green), or from fossil sources (black)) plays a role.[^7a^, ^29^] In the more recent literature, this criticism has led to increased scrutiny of life‐cycle considerations for CO_2_‐based processes. A fundamental examination of the hydrogenation of CO_2_ has been reported by Bardow et al.^[11d]^

The selection of a reaction for a chemical battery is currently in no way straightforward. High‐level discussions surrounding the structures of sustainable energy regimes currently include critical considerations of the use of chemical batteries. Unfortunately, it must be stated that none of the reactions discussed above have been tested in the form of a chemical battery for the storage of hydrogen with charging, transformation, and discharging on the scale required for a global technology. In Table 2, several viable molecules are listed along with parameters for their suitability as chemical batteries.

**Table 2:** Storage processes for chemical batteries.

Entry	Storage molecule	Mol H_2_ stored	Mol H_2_O lost	Storage density, gross weight [%]	Storage density weighted [%]
1	formic acid	1	0	4	4
2	urea	2	1	7	3
3	methanol	2	1	13	6
4	methane	2	2	22	5
5	dibenzyltoluene (LOHC)	9	0	6	6
6	ethanol	2	1	6	3
7	ammonia (comparison)	3	0	21	21

In addition to the simple stoichiometric parameters based on CO_2_, the central quantity for battery applications—the storage capacity—is given with a negative weighting to take into account the loss of hydrogen in the form of water molecules during the conversion of CO_2_. Consideration of these weighted capacities is quite sobering for all the CO_2_‐based processes. The comparatively favorable values for ammonia (Table 2, entry 7) underscore this fact.

As mentioned at the outset, however, it cannot be concluded that CO_2_ chemistry is unsuitable for chemical batteries. In addition to the data shown in Table 2, a comprehensive evaluation of chemical batteries also includes numerous technical and economic factors as well as, perhaps most importantly, system service of chemical batteries within an energy regime. The central factor is the use of the energy supplied by the chemical battery and, with it, the total benefit to the system. In this case, despite the unfavorable storage characteristics for hydrogen, carbon‐based storage systems are often preferred due to their high energy density as well as their management and use, both of which are technologically well‐understood.

With regard to the technological maturity of the processes in Table 2, it is apparent that the storage of hydrogen (molecule synthesis) is being intensively investigated. The following detailed section on methanol molecules will illustrate these activities. In comparison, the discharge process of the chemical battery, with the goal of recovering pure hydrogen (dehydrogenation), has enjoyed significantly less research and is not as well understood. This notable discrepancy arises from the lack of insight into the function and use of chemical batteries in a circular economy of energy carriers. An example is the LOHC process (Table 2, entry 5). At the other end of the spectrum is the reforming of methanol, which has been investigated intensively in the context of fuel‐cell automobiles.

## 5 Status of the Literature

**Figure 2:**
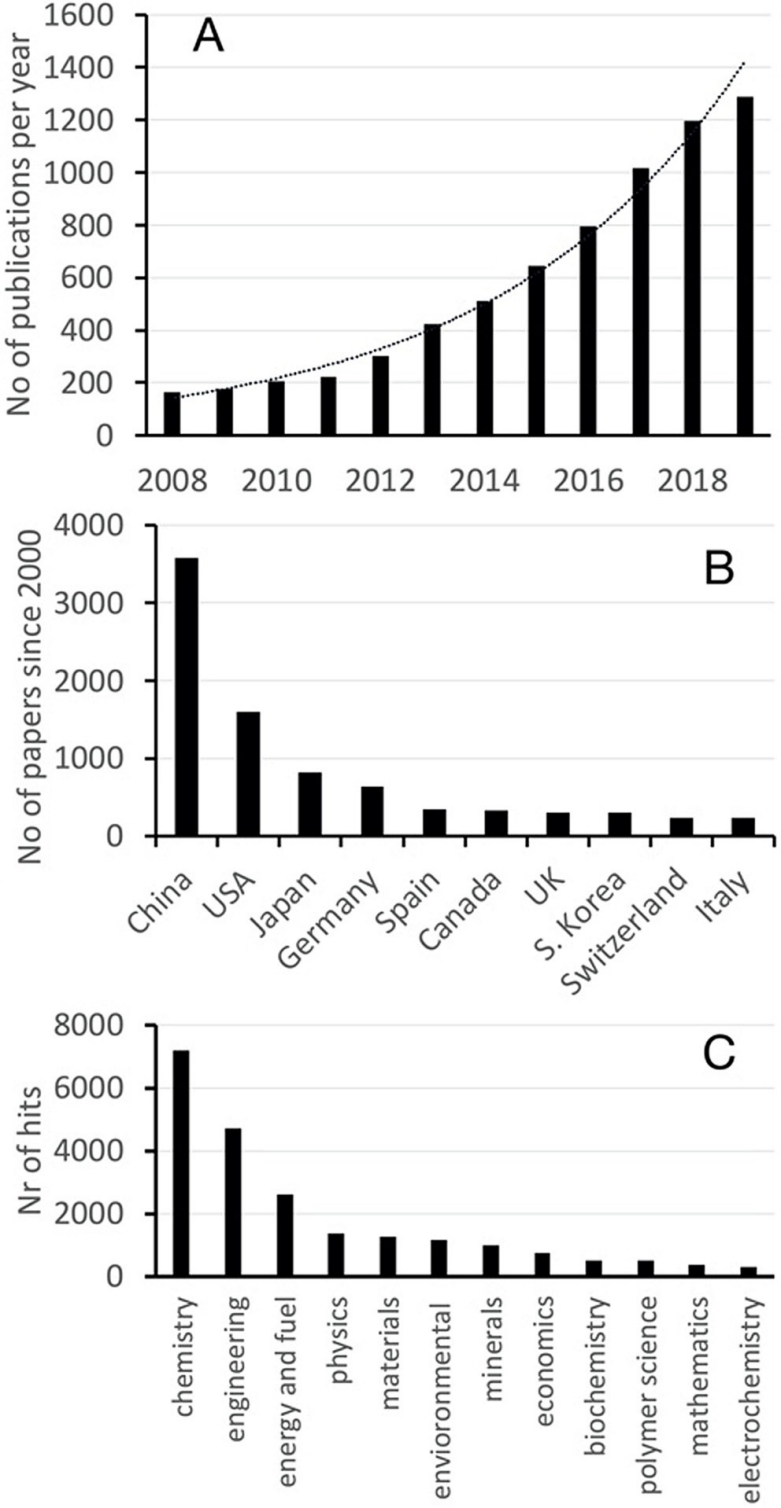
A) Number of publications on the topic of “hydrogenation of CO_2_.” B) Classification of publications by country. C) Classification of research fields appearing in *Web of Science* for the publications shown in (A).

Figure 2 summarizes key parameters from a literature analysis with the keyword “hydrogenation of CO_2_.” Ten years ago, about one publication on the subject appeared per working day. Today, that number is a factor of eight higher and is growing at an exponential rate. If the studies are sorted according to the reaction products, 80 % of all papers contain the four molecules methanol (53 %), methane (17 %), higher alcohols (16 %), and alkanes (14 %). The analysis underscores the view that the hydrogenation of CO_2_ is specifically geared toward the preparation of synthetic fuels.

If the analysis is performed according to the country in which the research was performed, it can be seen in Figure 2 that China maintains the dominant position. The countries traditionally strong in chemical research follow. In Europe, Germany is the leader, while the topicality of the subject in Switzerland is notable. If the classification of research fields from *Web of Science* is used (Figure 2 C), the topic is, of course, deeply rooted in chemistry, although a wide disciplinary range is found when carrying out this search. Furthermore, it can be seen that contributions to research on the hydrogenation of CO_2_ to generate commodities now come from fields not originally involved in finding solutions to these challenges.

In summary, CO_2_ hydrogenation is a current field of research and enormously dynamic, with the significant dominance of Asia being one of the main drivers. The subject is also investigated and treated in an interdisciplinary fashion. These conclusions are not surprising considering the substantial effort by China and Japan to reduce their reliance on imported oil. Further contemplation of the subjects to which the largest number of studies are dedicated reveals the notable difficulties remaining after a long time of investigation—the easy insights have already been gained. For this reason, the interdisciplinary approach is also of no surprise.

These considerations skip over many novel approaches stemming mainly from molecular chemistry and catalysis. The task of producing something chemically valuable from carbon dioxide has resulted in much creativity^[11a]^ and has led to a rich synthetic chemistry of CO_2_. How these results will mature into new approaches for the energy question cannot be answered at present. The necessary focus on the technologically significant questions of the day pertaining to the synthesis of known solar fuels is accompanied by the dangerous tendency for a narrowing of the “research pipeline” needed for the solutions of tomorrow as well as for innovative developments in the synthesis of intermediates and fine chemicals. Thus, the author emphatically supports pursuing novel approaches in the chemistry of CO_2_.[^13^, ^45^] Even if this goal, in contrast to some statements in the introductions of publications, does not lead to relevant contributions to the climate problem, such studies provide urgently needed in‐depth and fundamental insight about the reactivity of this important molecule. The result will be a library of synthetic options, which could be decisive for future material family trees in the chemical industry that are structured differently from our present reliance on petrochemical raw materials. This assessment seems also valid for the energy supply in the mobility sector. The immense size of this application^[7a]^ makes it difficult to implement novel concepts at a large scale for improved combinations[^49f^, ^53^] of fuels and motors. However, the large scale should not inhibit the search for better concepts. If research is allowed to cast this wide net in close coordination with the sciences involved, significant advances in the efficiency of the use of synthetic fuels[^7a^, ^50^] should be expected along with a reduction in the carbon leakage of the corresponding cycles. To hope for universal e‐mobility is likely short‐sighted in view of the extensive use of mobility in our societies.

## 6 Scale Effects and Urgency

**Figure 3:**
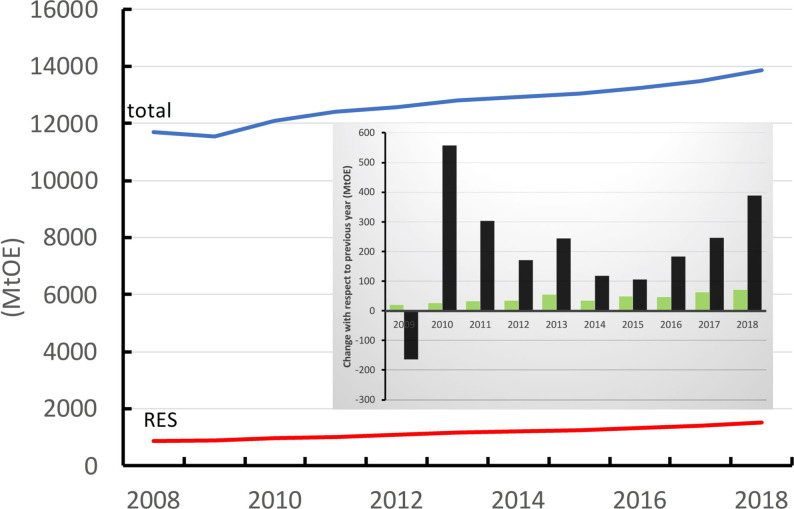
Development of a global energy supply. The red line shows the growth of all forms of renewable energy (hydro, biomass, wind, sun). The inset shows the annual growth in energy consumption divided into fossil (black) and renewable (green) sources. (Source: BP Statistical Review of World Energy 2019, 68th Edition.)

In nearly every publication, the desire to contribute to the energy transition is given as a source of motivation for the study. Several basic principles are, however, often overlooked in describing this impetus. The reason for the disconnect with real needs results from the fact that the defossilization of the energy regimes of the world is of extreme urgency and requires chemical conversion on an enormous scale. Figure 3 summarizes several quantitative arguments to this end. In the main graph, the development of global energy consumption is shown along with the fraction of renewable energy. It should be kept in mind that the vast majority of the latter stems from biomass (firewood) and hydropower. The “new energies”, wind and solar, which are the ones with the potential to supply the world, contribute to approximately half of all renewable energy. It is quite worrisome, as is shown in the inset of Figure 3, that the annual growth in fossil energy consumption is still significantly higher than the growth of the contribution from renewable energy.

The dimension of this growth is, at several hundred million tons of oil equivalent, certainly formidable, especially when considering that this number, if multiplied by three, is the number of supertankers required to transport this amount of energy around the world. The urgency with which chemistry must react is evident from these dimensions. Without the complementary use of renewables to produce a solar fuel energy cycle, it will hardly be possible to replace fossil fuels in the mandatory short time frame. With respect to the question of CO_2_ hydrogenation, only materials and processes should be considered which can be upscaled to an order of magnitude of 100 Mt/annum CO_2_ uptake and which rely on resources available in sufficient quantities. A huge number of large‐scale production facilities will be required to process such dimensions of CO_2_, the construction of which will take years. Solutions will have to be found in these facilities for fundamental problems resulting from the combination of intermittent green energy and the use of novel material cycles.^[54]^ Considering these factors, it is evident that one focus of research must be on the adaptation of existing processes to new challenges. Completely new approaches are important and provide more security for our future, but they should not be motivated by possibly contributing to the achievement of the goals of the Paris Climate Agreement. In general, it would help to orient the reader if the authors of studies dealing with these issues provided estimates on the scaling of their work with respect to material availability, robustness of synthesis processes, and feasibility. Subsequent studies that wish to deal with the actual implementation of proposed processes will then be better equipped to differentiate between urgent applications and solutions which will be relevant in the distant future.

This argument can be elucidated with an example. In the project C2C,^[54]^ an emissions reduction process has been realized during steel production by using CO_2_ hydrogenation with green hydrogen. The energy usage of the facility (Thyssenkrupp steel mill, Duisburg) is approximately 54 TWh/annum, or about 10 % of Germany's total consumption of electricity. In this project, only currently existing technologies can be employed to at a minimum partially redirect emissions into chemical applications in the next 10 years. Nevertheless, significant challenges appear during these operations in connection with the realities of fluctuating primary renewable energy sources. These challenges lead, with respect to opportune economical solutions, to fundamental questions about the dynamic operation of methanol synthesis or the configuration of minimal gas purification methods which do not harm the subsequent catalytic processes and also leave the products free of unwanted trace substances. The argument that other processes may also be involved in the reduction of emissions in heavy industry is taken into account in the C2C project by diversification of the product portfolio to include urea, higher alcohols, and reactive intermediates from CO_2_ as well as in using unavoidable CO_2_ sources such as lime production and waste incineration.

## 7 The Reaction Network

**Figure 4:**
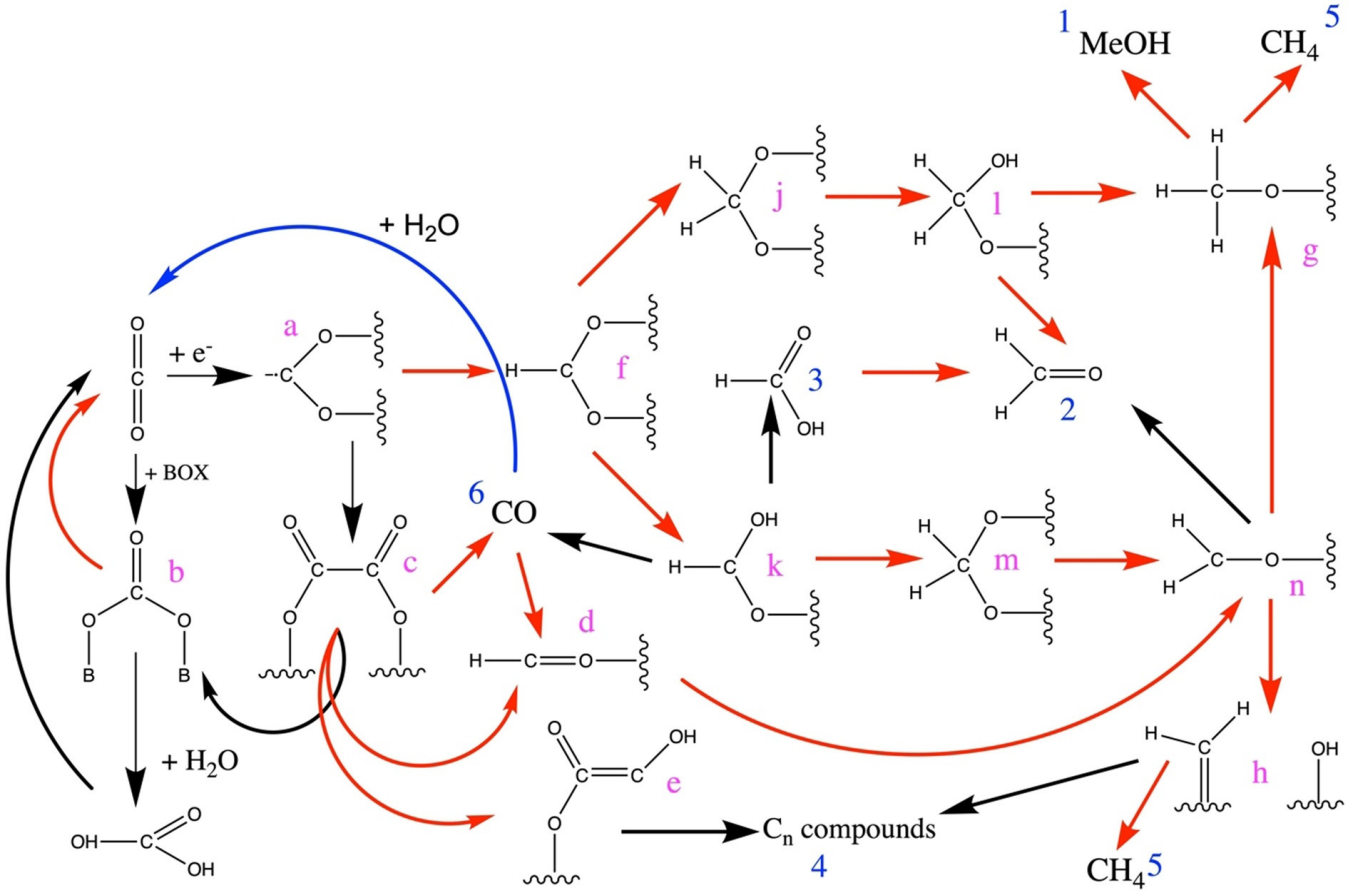
A section of the reaction network for the reduction of CO_2_ to simple products. The red arrows denote reduction reactions, the black arrows are redox reactions. The blue arrow shows the water–gas shift reaction. Stable products are labeled with numbers (blue), intermediates with lower case letters (red).

The activation^[11b]^ of CO_2_ begins with the transfer of negative charge to the initially linear CO_2_ molecule. As a result, the molecule becomes bent^[43i]^ and the stability of its electronic structure is lowered. Now, reduction products can be formed through reactions with hydrogen, or carbonates can be formed by reactions with a base. Figure 4 shows that further products are also possible through individual reactions.

It is apparent that the conversion of CO_2_ with hydrogen results in the accessibility of a large number of molecules which are both valuable for chemistry and suitable for renewable energy storage. The energy is delivered via hydrogen. A common complaint in the discussion on the production of resources from CO_2_ is that the processes are too “energy hungry.” All reduction reactions produce water, with the exception of the formal addition of hydrogen to CO_2_ to form formic acid (**3** in Figure 4). The water, with its high enthalpy of formation, provides the driving force for the endothermic target reaction. This process can be described as the charging of the “chemical battery” CO_2_, which can then be discharged in the subsequent oxidation reaction. It would, therefore, be ideal if—parallel to the necessary production of water—as much of the reaction enthalpy as possible could be stored in the target molecule (see also Table 1). In the case of formic acid (**3**), no water is produced. The “storage effect” is smaller in this case—but the energetic losses are also lower. For this reason, the formic acid molecule is also a valuable storage medium[^11e^, ^55^] and optimization of charging and discharging processes with suitable catalysts—if possible, without the use of noble metals—is a desirable avenue of research.

From Figure 4 it can be seen that the activation of CO_2_ initially involves a series of acid–base reactions. Carboxylate (**a**) can either dimerize[^43i^, ^56^] (a reaction often missed in the literature) to produce oxalate (**c**) or it can react with a Brønstedt or Lewis base (BO_x_) to produce carbonate (**b**). If water is added, carbonic acid is produced, which, if it is not esterified, will decompose into CO_2_ and water. The process can proceed spontaneously or with metal catalysts which poorly bind atomic oxygen.^[56]^ If an oxophilic metal such as Cu is used, oxalate can decompose into carbonate and CO (**6**). If hydrogen is present, H‐CO (**d**) is easily formed and can, via **n**, react to produce methoxy compounds (**g**) and methanol. The reaction need not proceed along the “formate,” or “reverse water‐gas shift” (RWGS) route (**j**, **k**). This fact is important, because it means a mechanistic pathway to methanol is available, which is consistent with the stepwise hydrogenation of CO and yet contains the carbon atom from the CO_2_ molecule. Isotope marker experiments designed to explain the reaction pathway of methanol synthesis are, therefore, ambiguous. A precondition for this reaction pathway is that the generated carbonate decomposes back to CO_2_ to close the catalytic cycle. This is the case for the catalyst system Cu/ZnO under the reaction conditions, as was shown through in situ investigations on the formation of this catalyst.^[59]^ The role played by water and protons^[60]^ in the formation of the intermediary carbonic acid is not currently known. One possibility is the formation of formaldehyde (**2**) from the intermediate (**n**). This process does not take place under normal reaction conditions because the additional energy of the subsequent reaction to generate methoxy compounds (**g**) is significantly higher than the energy maximum[^47i^, ^58^, ^61^] marked by free formaldehyde. The blue arrow in Figure 4 denotes the water‐gas reaction. There are different mechanistic views of this reaction that describe how lattice defects in the catalyst play an essential role in the redox reactions. For clarity, these reasons are not given here.

The reaction pathway[^21^, ^47i^, ^60^, ^62^] to methanol is usually described as proceeding from carboxylate (**a**) to formate (**f**). From this point, the process splits and results in the hydrogenation of either the carbon (**j**) or oxygen (**k**); the latter can lead to the production of formic acid (**3**). The intermediates **j** and **m** are identical, which makes experimental differentiation problematic. It has been observed that both formate (**f**), when used as a precursor for the synthesis of the Cu/ZnO catalyst, and formate co‐adsorbed with oxygen on copper decompose at 473 K.^[63]^ This leads to the presumption that the important formate–oxygen intermediate^[60]^ appears on the catalyst surface for only a short time before reacting to form more stable products. After the reaction begins, it is the dominant intermediate on the catalyst surface and can be readily identified as long as low reaction pressures are used.^[64]^ However, other adsorbates appear under pressures common in technical reactions.^[65]^

Under the conditions of gas‐phase catalysis, methanol (**1**) is formed from intermediate **g**, which, in the presence of acid sites, reacts further to generate dimethyl ether. Selectivity for methane (**5**) has not been observed under normal conditions with a copper catalyst. Under the conditions for electroreduction,^[8a]^ however, methane can indeed be formed, although the reaction is usually unwanted. Mechanistically, it is unclear whether methane is formed from intermediate **g** or via intermediates **n** and **h**. In contrast, the reactions between intermediates **h** and methanol to generate higher alcohols^[66]^ are very much desired. Alternatively, higher alcohols can also be formed along with hydrocarbons from intermediate **e**. To observe these reaction products on copper, on the other hand, co‐doping with a transition metal (oxide) or electroreduction is needed. The innumerable resulting reactions, when starting from CO or methanol,^[45]^ will not be covered as they are beyond the scope of this work. However, the chemistry of the Fischer–Tropsch synthesis^[67]^ must be mentioned in the context of CO and chemical energy conversion. This process results in the availability of diverse products and mixtures which are important as fuels. The industrial facilities for gas‐to‐liquid (GTL) processes make use of this chemistry in a modern form. Methanol itself^[68]^ can be used as a fuel, although it possesses unfavorable characteristics.^[69]^ In the future, therefore, the acid‐catalyzed oligomerization of methanol (methanol‐to‐olefins (MTO) process, or methanol‐to‐gasoline (MTG) process) will likely be used for fuels, or methanol will be etherized with formaldehyde to form oxymethylene ethers (OME)^[70]^ which are well‐suited to replace fossil diesel fuel. Methanol itself would be a cheap fuel as its synthesis pathway in a single step from CO_2_ is highly efficient; it would require, however, the design of a dedicated combustion process to avoid its blending with other molecules, which diminishes the synthetic advantage. The combustion of pure methanol can be very clean and minimize local emissions.

## 8 Methanol Synthesis

From the large number of possible reactions for the hydrogenation of CO_2_, the methanol synthesis reaction will now be considered in greater depth. In addition to the great significance of this reaction, it also serves as an illustration of the insight and uncertainties which exist today with respect to the function of a highly successful catalyst. The reaction network in Figure 4 provides the basic principles for kinetic models that precisely describe the technical synthesis of methanol. An assessment of these mechanisms^[71]^ has determined that the nature of the catalyst has little effect on the speed of methanol formation as long as the Cu/ZnO system is maintained. With this consolidated information, it should be possible to estimate the likelihood of finding a new catalyst^[72]^ or a novel process in this field which can be upscaled to the needs of energy applications.

Today, methanol is produced industrially from synthesis gas and hydrogen. Since the proposals of Asinger^[5a]^ and Olah,^[5b]^ the basic idea for defossilizing the energy system has been to replace the synthesis gas with CO_2_ driven in a cycle (the discharged state of the chemical battery) and to “charge” the battery with hydrogen from renewable sources (“green hydrogen”). Concern with respect to this concept has been expressed^[73]^ that the efficiency of technical catalysts based on Cu/ZnO/X would suffer if CO_2_ were chosen as the source of carbon instead of synthesis gas.

However, the situation is more complex if the reaction is made to proceed in a way that achieves a maximum space‐time yield. In this case, the catalyst operates near an equilibrium line which is defined through a network of reactions [Eqs. 6–8].

(6) $$\mathrm{CO}{}_{2}+3\;H{}_{2}\to \mathrm{CH}{}_{3}\mathrm{OH}+H{}_{2}O$$

(7) $$\mathrm{CO}{}_{2}+H{}_{2}\to \mathrm{CO}+H{}_{2}O$$

(8) $$\mathrm{CO}+2\;H{}_{2}\to \mathrm{CH}{}_{3}\mathrm{OH}$$

Thus, under the conditions of high CO_2_ conversion, there is always also a partial pressure of CO and water in the catalyst bed. If pure CO is used as the initial molecule, very harsh conditions are required (BASF process) to produce methanol.^[74]^ Today's common Cu/ZnO/X catalyst was developed for the purpose of producing methanol[^74^, ^75^] from CO_2_. In this process, the multifunctionality of the ZnO components^[76]^ play the different roles of carrier, mineral stabilizer of nanostructures,^[77]^ and co‐catalyst^[78]^ on copper. The formation of a Cu‐Zn surface alloy[^47h^, ^79^] as the active phase has also been the subject of speculation and attempts have been made to verify it experimentally. Furthermore, the Cu/ZnO/X catalyst is well‐researched in terms of the structural dynamics^[47j]^ of the active phase. The resulting classification of structures and functions has turned out to be complex, because different active forms of the catalyst exist under the distinct conditions of any particular investigation.

If the fact is considered that the conversion into methanol is restricted under practical, relevant conditions,^[74]^ the product gas must be recycled over the catalyst after water and methanol have been separated out. This procedure ensures that the catalyst is exposed to all three equilibrium reactions [Eqs. (6)–(8)], even if a pure CO_2_/H_2_ input gas is used. In technical processes, the synthesis gas can be chosen such that only small amounts of water are produced to protect against possible decomposition of the delicate nanostructures of technical catalysts. It should be noted that a catalyst decomposition through reduction and resulting in brass^[59]^ is also harmful and explains why a certain amount of water is required^[80]^ to stabilize the system. Current studies (still in progress^[81]^) show, however, that the harmful effect of water depends significantly on the absolute pressure as well as other contaminants (notably traces of oxygen) in the input gas present during the reaction.

**Figure 5:**
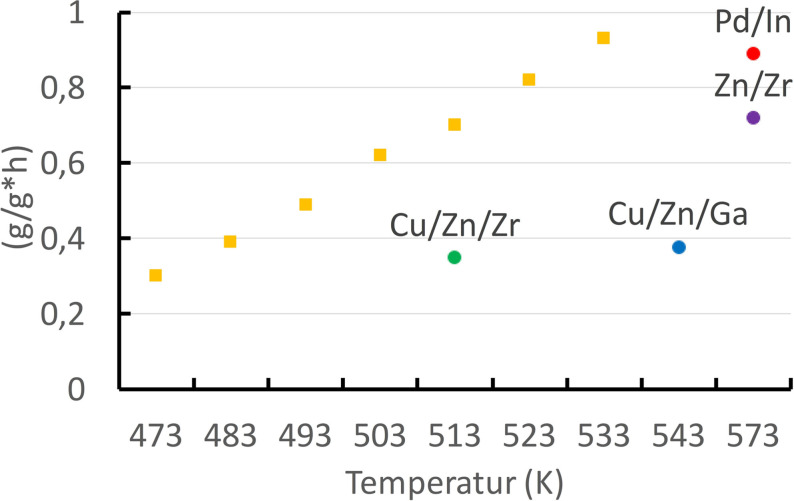
Space–time yields for methanol over a technical Cu/ZnO catalyst as a function of temperature (yellow squares). Experimental conditions: 30 bar, 1:3 CO_2_/H_2_ mixture^[82]^ (the Zn/Zr system was measured at 50 bar). The individual data points for selected reference systems were taken from a literature review.^[83]^

The performance of technical Cu/ZnO catalysts during the reduction of CO_2_ to methanol has been determined as a function of temperature. Figure 5 shows that technical catalysts can indeed achieve a direct hydrogenation of CO_2_ with a relevant space‐time yield.

Figure 5 also demonstrates that other experimental catalytic systems^[83]^ are not discernibly superior to the Cu/ZnO system in terms of their productivity. Further reports detail that the Cu/ZnO/ZrO_2_ system can exhibit noticeably increased productivities of up to 1.2 g g^−1^ h if the space velocity is increased by about a factor of 10 compared to normal values.

The relatively marginal sensitivity of the reaction to the nature of the catalytic system^[71]^ is a consequence of the multiple equilibrium reactions which ensure that CO and CO_2_, along with water, are present along most of the length of the catalyst bed. It is, therefore, not strictly necessary[^47b^, ^58^, ^71^] to develop a new catalyst for the reduction of CO_2_ to methanol because the performance and stability of the Cu/ZnO system approach the reference values under synthesis gas conditions. It seems possible to influence how completely the equilibrium is reached through modification of the composition of the Cu/ZnO system. Systems with large amounts of ZnO^[85]^ result in high methanol yields and considerably less CO than would be expected from the position of the thermodynamic equilibrium. Hence, the possibility may be considered that the selectivity of a catalyst toward the normally unwanted CO can be influenced to some extent while the productivity remains constant. The reason for this is presumably^[74]^ that effective water‐gas reactions at the defects of ZnO results in a higher conversion than the reverse reaction at the copper sites. The copper is also present on the catalyst surface, but is expected to exhibit a lower reactivity than the ZnO. Such arguments tacitly suppose that a distribution of active sites exist on a working catalyst, thus allowing the possibility to manipulate the extent to which the pathways shown in Figure 4 are followed after activation of the duct molecules.

As a consequence of the position of the chemical equilibria between CO, CO_2_, and hydrogen, it would be desirable to increase the catalyst activity so that methanol synthesis can take place at temperatures below 473 K, where methanol is in a liquid state (slurry).^[86]^ For this, the development of a catalyst completely different from the copper‐based catalyst is likely required. It has been shown for the Cu/ZnO system that an adsorbate layer of water, ‐OH groups, as well as formate and methoxy intermediates blocks the active centers^[87]^ during methanol synthesis as soon as the temperature falls below 483 K. The lower limit of the working temperature of this system is, therefore, determined by the reaction products preventing access to the active centers and not by an intrinsic, insufficient activity. This shortcoming could be rectified if it were possible to find an active catalyst that could perform without the synergy[^77a^, ^88^] of copper and zinc oxide. One source of motivation for this search could be the use of catalysts which can produce methanol from CO under mild conditions. The MoP system has recently been introduced^[89]^ and may provide a clue to the correct approach. Furthermore, it is important for any successful application that no methane is generated as a by‐product. The Cu‐based systems[^66c^, ^66d^, ^90^] display these characteristics in the gas‐phase hydrogenation of CO_2_ and CO.

## 9 Copper as a Catalyst

The element copper has achieved a distinguished position in the catalytic reduction of CO_2_ due, among other things, to its ability to catalyze a gas‐phase reaction with a high selectivity for the production of methanol. If solid acids are present, the reaction can also produce dimethyl ether.[^83^, ^91^] In particular, the process does not seem to produce any C−C bonds, although this is certainly possible according to the reaction network shown in Figure 4. This behavior is often ascribed to the electronic structure of copper (see discussion in Ref. [89]) and it is expected that catalysts with a partially occupied metallic d band will be required to produce higher hydrocarbons or their oxo derivatives from CO_2_ (CO). It is certainly possible to modify copper with co‐catalysts to “toggle” it into catalyzing the formation of C−C bonds.^[92]^ However, it is unclear whether this is a tandem reaction of CO production followed by the conventional hydrogenation of dissociated CO on a co‐catalyst, or methanol generation followed by carbonylization with the simultaneous production of CO or, finally, the direct formation of a C−C bond during the reduction of CO_2_.[^33b^, ^93^] These kinds of considerations are not only pertinent to copper, but also, for example, to rhodium‐based systems.^[94]^ Here, however, the necessary CO must be produced in a preceding reaction and, in addition to oxygenates, methane and other hydrocarbons are co‐produced.

The observation^[8a]^ that the electrochemical reduction of CO_2_ with copper can lead to an entire series of hydrocarbons is, therefore, even more striking.[^47e^, ^95^] This result is also found with other transition metals,^[96]^ although they can also generate such products during gas‐phase catalysis. An oxidative pretreatment of copper also seems advantageous[^47e^, ^97^] for the production of higher hydrocarbons and their oxygenates.

It can be concluded from these considerations that the branching of the reaction network in Figure 4 is determined by the state of the surface (chemistry and morphology) of the catalyst. The excellent selectivity of Cu for the formation of methanol under high‐pressure conditions with hydrogen‐rich reaction gases, only small amounts of water, and temperatures between 473 and 573 K is evidently due to the formation of a unique chemical state of metallic copper (“methanol copper”). Another state of copper is formed if conditions are shifted to those of electroreduction in an alkaline electrolyte at 300 K and a reaction potential capable of reducing Cu‐oxide mixtures, which are stable when not in the presence of an electric current. Common to both states is that they consist overwhelmingly of metallic copper. The differing reactivities show, however, that the chemical and structural states are apparently not identical. The importance of the pre‐history of an activated Cu surface illustrates that the states do not correspond to thermodynamically stable phases and that chemical dynamics^[98]^ dictate the exact nature of each state. The electrolyte also seems to play a significant role^[95b]^ in the process.

The different chemical states of the copper metal can be understood by remembering that electrochemical reduction takes place at room temperature and diffusion of oxygen atoms is slow. Rapid and deep diffusion is, however, very much a possibility^[99]^ during the activation of the gas‐phase catalyst.^[59]^ Furthermore, the reactivity of hydrogen is different along with its redox potential with respect to oxygen in CO_2_ and to the intermediates in Figure 4. Although atomic hydrogen is assumed in gas‐phase catalysis, “nascent hydrogen” with a hydride‐like electron configuration and a significantly stronger hydrogenating effect can arise by the electrochemical reduction of water. In addition, the residence time of the intermediates on the electrocatalyst may be longer than on the gas‐phase catalyst. For this reason, complex reaction sequences have a higher probability in electrocatalysis.

Comprehensive studies on the selectivity of Cu metal in differing preparations as well as Cu oxides and alloys are described in an exhaustive review on the electroreduction of CO_2_ with molecular catalysts at interfaces.^[46a]^ Unfortunately, most of the systems described therein are extremely complex in their interface chemistry, and trends are difficult to identify. The different reaction pathways shown in Figure 4 are certainly all represented. With improved^[8a]^ measurement precision, the various crystal orientations in an electrode can be differentiated electroanalytically and in situ.^[100]^ The effects described above of the impact on product selectivity through different electrochemical pretreatments of a copper surface can, according to rigorous spectroscopic investigation,^[95b]^ be linked to a combination of the presence of a surface copper oxide^[97d]^ and morphological effects (“roughening”). It seems that the production of C_2_ compounds can be traced to the surface oxide,^[101]^ whereas copper modified at the surface by oxygen without oxidation^[102]^ is beneficial for the production of C_1_ compounds.

This hypothesis differs from an assumption in gas‐phase catalysis that adsorbed atomic oxygen is necessary as an oxidant for CO in the synthesis gas to enable formate intermediates. Pulse kinetic measurements have clearly shown^[43h]^ that no appreciable concentration of reactive atomic oxygen is present under the conditions for gas‐phase methanol synthesis. In contrast to this, it has been known for some time that a copper electrode binds oxygen at its surface^[103]^ at all pH values, although its protonation to generate OH groups depends on the pH value and applied potential. The thickness of a modified termination layer on such an electrode has been estimated to be 10 monolayers.^[103]^ The electrochemical oxidation of copper results in a likewise thin passive layer^[104]^ consisting of a mixture of CuO and Cu_2_O.

The question of the chemical nature of the active copper has long been the subject of investigation.[^47c^, ^47h^, ^61^, ^77a^, ^79^, ^83^, ^97a^, ^97b^, ^105^] It is indisputable that all activated copper catalysts and electrodes overwhelmingly contain metallic copper in the bulk phase.^[106]^ Less clear, however, is whether this copper is pure.^[99a]^ Here, “pure” means that the copper, when active, contains neither zinc as an alloy^[107]^ nor residues of oxygen from preceding states^[106b]^ (either as an oxide or dissolved in the bulk^[108]^). Furthermore, pure means that the active copper is not involved in a “strong oxide–metal interaction” with a surface phase of ZnO. There is evidence for the formation of a strong metal–support interaction (SMSI) surface layer.[^78a^, ^105e^, ^109^] Other studies (under ETEM conditions) cannot identify the layer with certainty.^[110]^ In the latter case, however, different catalysts were investigated (ratio of carrier/Cu and microstructure/carrier). It has been shown[^88a^, ^106a^] that the interaction energy between Cu and ZnO differs depending on the degree of reduction of the ZnO. This interaction can modify the morphology of a pure copper cluster. Defective ZnO_1−*x*_ can “creep” onto and around a Cu cluster that has been, for example, contaminated with and roughened by dissolved oxygen.^[61]^

It is questionable whether the functionality of copper in the electroreduction of CO_2_ can be further clarified if another element,^[111]^ such as Co[^66b^, ^66c^, ^66e^] or Ni, is added to the copper‐based catalyst because they change the reactivity in a complex way. The chemistry of the support is likewise critical. A very different reactivity of copper was demonstrated with respect to hydrogen depending on whether diamond or oxygen‐containing sp^2^‐hybridized carbon was used. With respect to the latter, a heterolytic H_2_ dissociation seems to occur.^[112]^ This conclusion was also supported by electron microscopy studies on Cu nanostructures,^[112]^ which identified a distinct corrosive action of hydrogen on carbon carriers of differing quality. The behavior was clarified by a “spill over” of hydrogen on graphene structures. On diamond carriers, on the other hand, hydrogen only reduced oxidic copper without corroding the carbon.

## 10 Methanol Copper: Structure and Dynamics

The interaction of copper and its carrier material is dependent on the chemical potential of the environment. This ensures that the Cu‐X system exhibits a distinct structural dynamic:[^88^, ^113^] the structure and wetting of the copper is changed reversibly as a function of chemical potential.^[110]^ Originally, it was even postulated that the active form of the catalyst contained copper dissolved in ZnO.^[114]^ A peripheral discussion concerns the question of whether active copper is affected by a carbonate phase[^59^, ^115^] that may act as a binder phase between the metal and its oxidic supports. In the case of electrolytic copper, there is the additional issue of the presence of OH or components of the electrolyte when the electrode is not thermally treated in an adequate manner after electrochemical synthesis.^[116]^ Furthermore, the molecular structure of the copper, that is, the type and number of lattice defects, plays a significant role in the reactivity[^61^, ^117^] which is not reflected in the statement “copper metal is the catalyst.” It has long been known^[118]^ that completely pure copper surfaces interact very weakly with adsorbates, while surfaces with co‐adsorbed oxygen exhibit a strong interaction. This function can also be taken over by dissociated water. For this reason, the separate branches of the reaction network for the gas‐phase reaction and for the electroreduction of CO_2_ (see Figure 4) may actually be determined by a different extent of hydroxylation^[119]^ of the metal surface with either water from the elelctrolyte or water from the reduction of CO_2_. The statement that “the catalyst consists of metallic copper with local, additional structural and chemical modifications” will, therefore, describe the reality of a working high‐performance catalyst much better than just stating that methanol copper is a metal.

**Figure 6:**
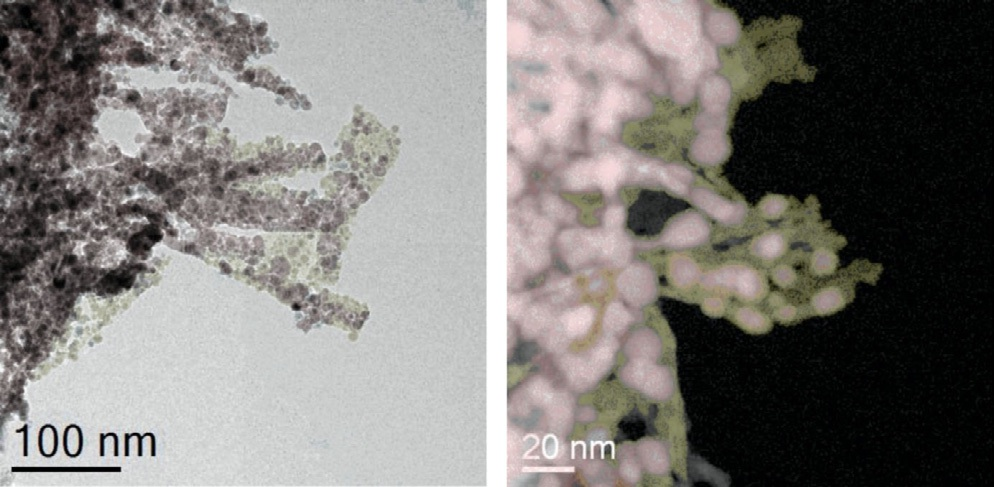
Morphology of a Cu/ZnO/Al_2_O_3_ catalyst produced with the aging method. The annular dark‐field (ADF) image on the right shows the ZnO (yellow) coverage of the Cu particles (pink).

To give an impression of the function lying at the heart of the active structure of a Cu/ZnO catalyst, the variety of morphologies will now be briefly discussed. The systems described were produced by co‐precipitation. Two kinds of catalysts, both with the same chemical composition, are obtained by controlling the kinetics of the precipitation and the subsequent work up. In one case, copper nanoparticles are supported on a porous mesh of ZnO needles. The nanoparticles partially coated the ZnO in a complex manner, while they themselves are partially coated by a reduced form of graphitic ZnO.^[121]^ This resulting structure is shown in Figure 6 at different scales.

**Figure 7:**
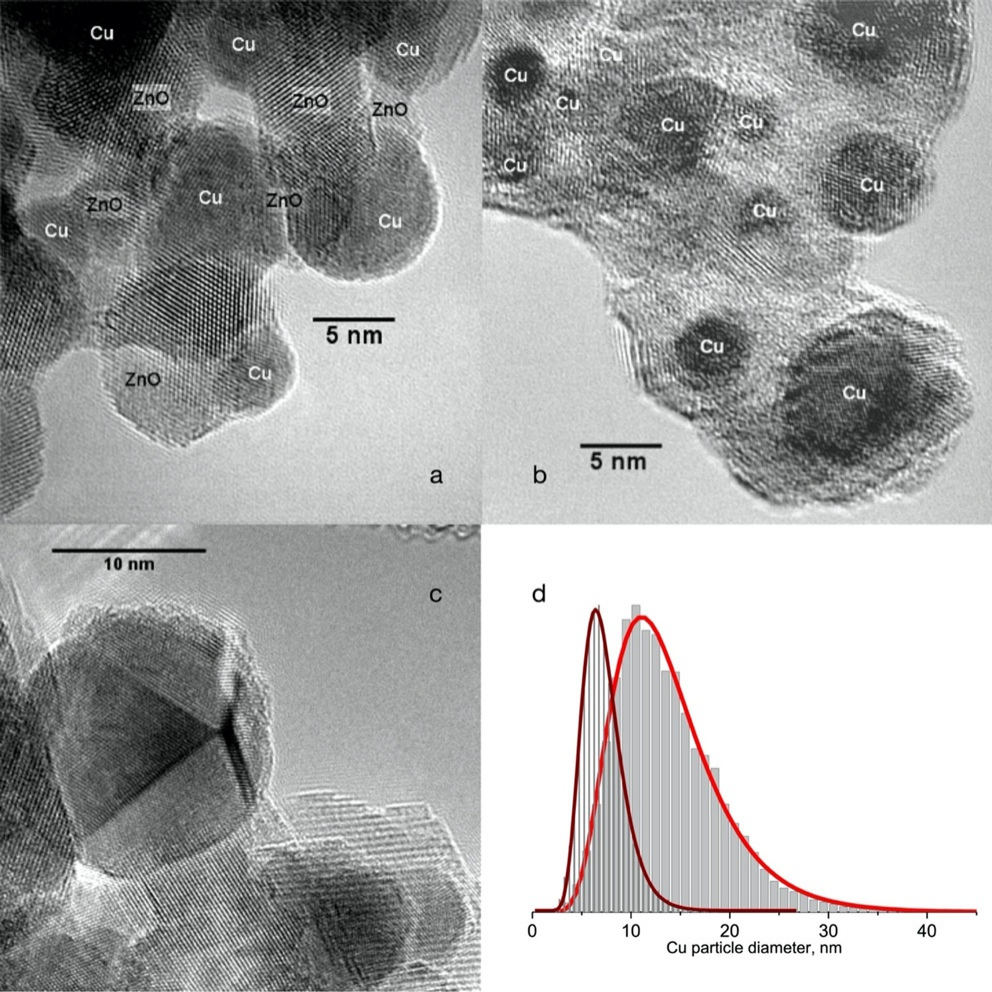
Morphology of Cu/ZnO catalysts. TEM images (a, b) of fresh samples: a) from discontinuous precipitation and b) from continuous overflow precipitation. c) Typical image of the catalyst in (a) after methanol synthesis at 10 bar in a CO/CO_2_ mixture at 503 K. The particle size distribution in (d) was generated from the samples in (a; narrow distribution) and (c; broad distribution). 5000 particles were analyzed for each distribution. Data taken from Ref. [122].

In the second case, the copper was embedded as nanoparticles in a ZnO matrix and resulted in a compact agglomerate of platelets. Although the exposed copper surface shown in Figure 6 is actually larger than that of the platelets, the latter result in an approximately 50 % higher activity per copper surface^[122]^ than the exposed particles. Figure 7 a,b shows TEM images of both forms of copper. High‐performance catalysts are composed of significantly more copper than zinc oxide, as is evident in Figures 6 and 7. This fact should be kept in mind during discussions of model systems, which often consist of only small amounts of copper particles on the carrier oxide. In particular, the ability of Cu to dynamically adapt its morphology to the reaction environment in high‐performance catalysts is markedly limited due to the high density of particles or to their fixed embedded position in the carrier oxide. Therefore, the dynamic adaption of the carrier oxides themselves plays an important role in these systems.[^77b^, ^123^]

From diffraction images and corresponding powder X‐ray data, it follows that the lattice constant of the exposed particles is, at 0.3617 nm, very similar to that of pure copper (0.3615 nm). In contrast, the embedding of copper in ZnO creates lattice defects in the copper, thereby resulting in a significantly higher lattice constant of 0.3625 nm.^[122]^ Figure 7 c shows a used catalyst of the type shown in Figure 6. After 400 h of reaction time at 10 bar it has aged and lost 20 % of its initial activity. Many twinned sinter particles of Cu plus platelets and amorphous fractions of ZnO can be seen. Figure 7 d shows the changes in the distribution of volume‐weighted particle size resulting from the aging process. This behavior has been reported many times in the literature and attributed to the harmful effect of pure CO_2_ as an input gas. However, improved synthesis methods lead to a better coating of the Cu active particles with ZnO so that the aging process is slowed and activities are mainly reduced through crystallization of the ZnO components^[123]^ resulting in them losing their protective function as well as their possible co‐catayltic function by a loss of interface area. The analysis and optimization of the catalyst is complicated by the role of lattice defects in ZnO.^[110a]^ The defects are caused by promotors and are responsible for the crystal's structural function^[124]^ as well as its activation of CO_2_ through electron transfer.

A richly featured nanostructure and a strong dependence of the reactivity on copper particles have been identified as important characteristics[^47e^, ^97b^, ^105c^] for controlling catalytic function during electroreduction. A complete and artifact‐free structural analysis is still ongoing.^[125]^ Re‐activating a given electrode surface structure back to its initial state by pulse voltammetry after short operating times was traced back to the restoration of a particular facet termination.^[125]^ Whether this interpretation is actually unambiguous is not fully clear when considering the many forms of atomic oxygen which also take part in the process.

The astonishing diversity of the answers to the seemingly simple question about the nature of the catalyst surely means that there are different forms of methanol copper. The reaction environment may be partially responsible for the variety of active states. In particular, the presence of oxygen in the many manifestations of activated catalysts plays an important role along with the non‐translational structure of the copper. These two factors are linked, because oxygen causes stress and strain in copper which results in a roughening of the copper surface. Additionally, an effect of the initial state of the oxide on the formation of the roughened surface is also relevant. The initial state (oxidation state, crystal form) is dictated by the activity of oxygen during formation of the preceding compound. For example, smooth metallic particles in all orientations can be formed from Cu_2_O crystallites by topotactic reduction. The structural motif of CuO, on the other hand, requires a non‐topotactic conversion where the roughening is a consequence of the formation of polycrystalline particles^[126]^ (crackling core and shell model). The demand to achieve a large active surface does not allow the normal chemical synthesis procedure to choose sufficiently radical reaction conditions to obtain a product at the thermodynamic minimum, for example, single‐phase, pure copper. In catalysis, very mild methods of precipitation,^[127]^ or impregnation^[128]^ and subsequent activation are used, which result in materials which are difficult to characterize. In the case of copper, there is the additional problem that the element, due to its pronounced affinity for oxygen, habitually tends to form solid solutions with oxygen, sub‐oxides,^[129]^ and surface oxides.^[97d]^ The situation is further complicated through the indirect effect of foreign particles on the internal stress and strain in the copper.^[130]^ This geometric defect state is consequential for catalysis because it changes the electronic structure of the surface (d‐band shift, step formation). The debate in the literature on the nature of the active phase is ambiguous, because it is unclear exactly which form of methanol copper was used in the different studies. Even rigorous measurements on single crystal copper do not prove that it is only metallic copper that acts as an effective catalyst. This ambiguity persists, despite the fact that single crystal copper actuates methanol synthesis and that the effect can be matched^[62]^ with data from a sophisticated kinetic model of a complex technical catalyst. The implication that all other forms of the catalysts are, in the best case, “contaminated” must be treated with great caution. This prudent approach is strengthened by descriptions of the material state of the activated state of copper, such as the analyses of surfaces with electron microscopy[^59^, ^132^] and state‐selective vibrational spectroscopy using CO as a probe molecule.[^109a^, ^133^]

The following overall picture of methanol copper during gas‐phase catalysis arises: in all cases, metallic copper forms the matrix phase—all analytical methods show this to be the quantitatively predominant phase. The simplest case is metallic copper with a roughened surface. The roughness is likely caused by internal forces of stress and strain;^[126]^ the roughened regions are stabilized by oxygen atoms and contain electron‐poor^[99b]^ Cu species but do not correspond to a crystalline oxide. Oxygen atoms (and/or OH groups) are rigidly bound at or in the surface, although they do not act as oxidants for CO. If the copper is supported by an oxygen‐containing substrate (oxides, carbon), or if it is partially embedded within an oxide matrix^[134]^ (ZnO, ZrO_2_, CeO_2_), perimeter states[^132^, ^135^] will be formed on which copper can interact with the oxygen of the substrate, thereby forming electron‐poor Cu species. A direct image of such perimeter states, which activate CO_2_ on an Au/MgO model system, has been published by the Freund group.^[56]^

If ZnO is present, it can be chemically reduced through very dry conditions or a high chemical potential of CO. The result is the local generation of a brass crystal structure.[^47h^, ^107^] Under the conditions of a finite conversion into methanol and water, the ZnO, as a defective (partially reduced) oxide phase,[^110a^, ^124^, ^136^] likely takes the form of a dynamic thin film^[109b]^ that is sensitive to the local chemical potential and covers the Cu roughened by internal stress and strain and by embedded oxygen atoms.[^61^, ^126^] A preformed brass phase would quickly be oxidized back to a defect‐containing ZnO phase^[105i]^ by water and/or active oxygen from the CO_2_ activation. Oxygen atoms are required for the partially reduced and porous^[137]^ defect ZnO film^[135b]^ to adhere to the copper at all. The oxygen facilitates the fixation of the zinc oxide layer (possibly with additional carbonate) especially well if it segregates from the bulk of the metal to the surface and forms a surface oxide.^[105i]^ ZnO is multifunctional—as the carrier for the Cu nanoparticles, as the active co‐catalyst, or as matrix phase for embedded Cu particles. The exact non‐translational structure of the ZnO, as a stoichiometric mineral separator, as a matrix phase, or as a defect interface layer (or as a combination of all these; see, for example, Figure 7 c), has been determined[^59^, ^110a^, ^136^] both by crystallization^[138]^ from the precursor carbonate compound (needles, platelets, orientation) and by contact with the reactants (hydrogen and/or CO). All Cu/ZnO systems, therefore, contain a perimeter line at which Cu and ZnO interact. The strength of this interaction depends on the exact redox state of both phases, which can vary according to the morphological orientation and the local chemical potential. It is probable that the perimeter line corresponds to the geometrical location of the active centers for CO_2_ reduction.

The existence of methanol copper can be verified, beyond the controversial discussion summarized here, by microcalorimetric experiments with CO and CO_2_ as probe molecules. For such an experiment, nanostructured Cu was synthesized by using ZnO doped with Al or Mg (3 % by weight in each case) using the conventional co‐precipitation method. As a comparison, Cu nanopowder was also produced through precipitation and activation.^[78a]^ Using the results obtained by Muhler and co‐workers^[105e]^ through calorimetry, the effect of ZnO as a carrier, which can be reduced easily (Al doped) or only with difficulty (Mg doped), on the chemisorption behavior of Cu will now be examined.

**Figure 8:**
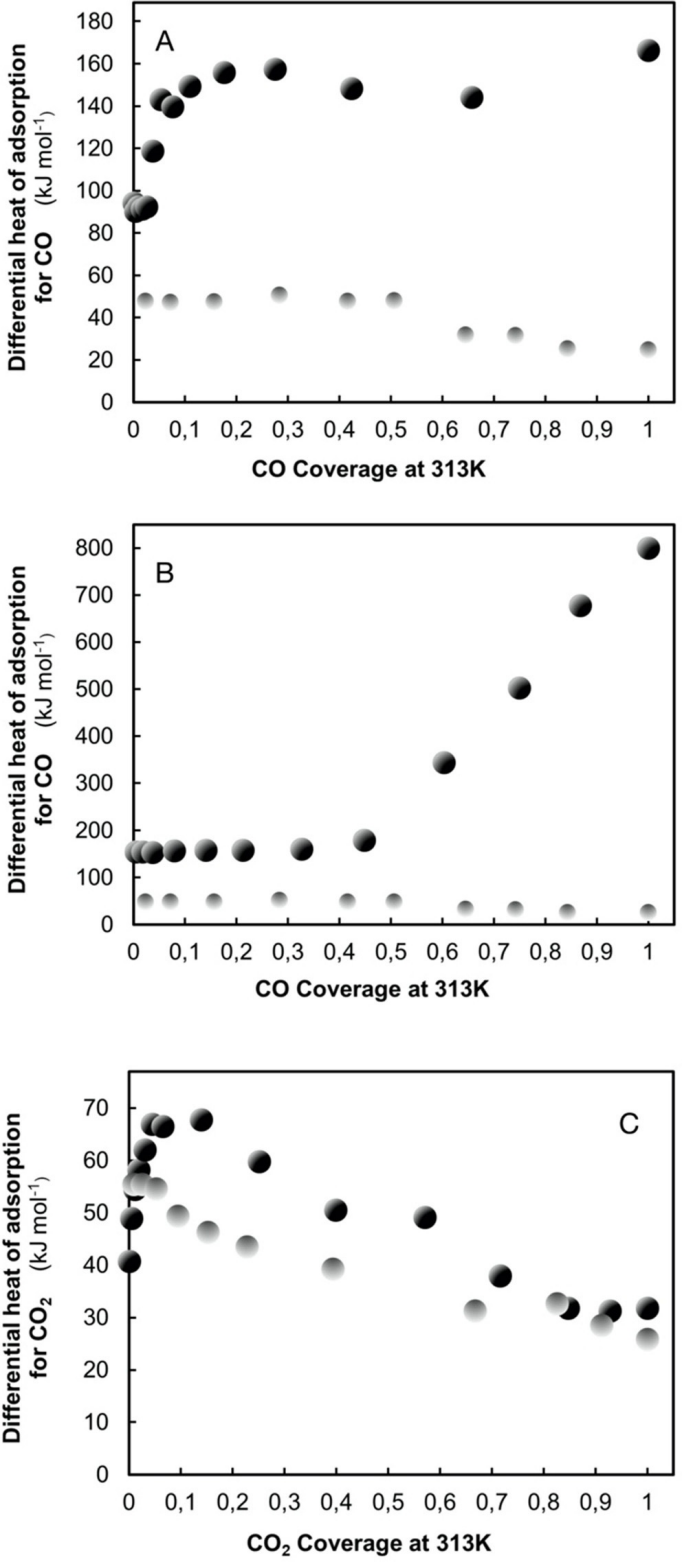
Adsorption calorimetry on promoted Cu/ZnO catalysts^[78a]^ after activation at 523 K in a 1:20 H_2_/N_2_ mixture. A) CO on Cu nanoparticles (lower line) and Cu/ZnO/MgO (upper line). B) CO on Cu nanoparticles (lower line) and Cu/ZnO/Al_2_O_3_ (upper line). C) CO_2_ on MgO (lower line) and Cu/ZnO/MgO (upper line). The units of the *x* axis were chosen to correct for the different geometrical surfaces of the samples and were calculated from the isotherms.

It is evident from Figure 8 that “pure” nanostructured copper adsorbs CO in a typical manner. The observed heat of adsorption agrees well with data from model systems.^[139]^ At a coverage of approximately 40 %, the adsorbate–adsorbate interaction begins to weaken the bond to the metal until, at full coverage, the approximate heat of condensation is reached. The case is completely different for catalysts on ZnO carriers. The adsorbate–catalyst interaction is significantly stronger than at the pure metal and suggests a substantially modified electronic structure of the “methanol copper.” The adsorption energy, which grows with the degree of surface coverage, can be explained through the structural dynamics^[110]^ of the copper and the accompanying development of new active centers. Although Muhler and co‐workers observed this phenomenon too,^[105e]^ they interpreted it as an artifact. A similar observation was also reported by Parris and Klier,^[105h]^ who explained it as the interaction of CO with electron‐poor copper or ZnO. If the adsorbate–adsorbate interaction increases to a coverage of approximately 0.4, a new phenomenon appears. The adsorbate begins to react with ZnO and causes the interaction energy to increase. This behavior can be seen clearly in Figure 8 B. A comparison of Figures 8 A and B shows the effect of the inhibiting promotor Mg on the reduction of ZnO in Figure 8 A and the activating effect of Al on the reduction of ZnO in Figure 8 B. The adsorption of CO_2_ on the Cu catalyst with inhibited ZnO reduction is shown in Figure 8 C. To exclude interference from the potential formation of MgCO_3_, pure nanocrystalline MgO was measured as a comparison. The behavior of CO_2_ and CO are largely the same with regards to the dynamic exposure of the adsorption sites. In the case of CO_2_, however, the absence of the reductive interaction between CO_2_ and ZnO results in the more expected behavior of gradually weakening adsorption.

## 11 Copper and Oxygen: A Unique Relationship

Molecular catalysts with copper show a diverse chemistry of interaction with the element oxygen depending on the different geometries and oxidation states.^[140]^ The diversity of the Cu‐O interaction is also present at the Cu surface. The geometrical environment (smooth, rough), the presence of oxygen below the surface, and the formation of oxide–metal interfaces in the bulk and on the surface are responsible for the distinct chemical forms and dynamics[^129b^, ^129c^] and can be identified using spectroscopic methods. If a possible co‐doping with Zn or another metal is included along with the other chemical possibilities, the result is a wide array of local electronic structures similar to the molecular systems and their complex ligand systems. The resulting diverse local electronic configurations govern the reactivity of the adsorbed CO_2_ and its products during a catalytic reaction. The quantitative determination of the reactive surface of the Cu/ZnO system provides an example. Unspecific adsorption of nitrogen generates the geometrical surface. Using other probe molecules with specific chemisorption results in significantly smaller “active surfaces” which can, as expected, be differentiated through selection of the probe molecule. Table 3 provides an indication of these effects.

**Table 3:** Surfaces in m^2^ g^−1^ of two Cu/ZnO catalysts determined by physisorption of N_2_ and by chemisorption of probe molecules.^[a]^

	Probe	Cu/ZnO/MgO	Cu/ZnO/Al_2_O_3_	
	N_2_	95	117	
	N_2_O	4	15	
	H_2_	3	13	
	CO	17	9	
	CO_2_	15	n.a.

[a] The conversion from adsorbed material quantities to surfaces should, for chemisorption, be viewed critically. Nevertheless, they were chosen here to give an impression of the relative orders of magnitude.

It is unlikely that pure copper is the only active catalyst, because both samples in Table 3 exhibit the same content of copper and the same nanostructure (particle size of 5–10 nm). The concept, that the catalyst has only a small effect on the formal kinetics of methanol synthesis^[71]^ is, however, understandable considering the dynamic control of the active surface by the chemical potential of the reactant mix. An assessment of the efficacy of the two catalysts according to the concept of a static turnover frequency^[142]^ is, in the best case, relevant with respect to the order of magnitude.

The ability of copper to stabilize multiple chemical states of oxygen is significant because the reduction of CO_2_ represents a case of redox chemistry. In the formal simple reaction [Eq. 9], two electrons are transferred in addition to the oxygen atom. It is clear that a redox catalyst such as Cu is required. The assumption that electrons are transferred through each adsorption center means that a metallic center having the ability to bind oxygen as an oxyl group would be advantageous. The formation and reactivity of the oxyl intermediate would then have to be explicitly considered in the context of actual microkinetics.^[143]^ The fact that no reactive oxygen was observed in pulse experiments^[43h]^ does not mean, according to the dynamic concept of the system, that a direct oxidation of CO cannot take place under the reaction conditions.

(9) $$\mathrm{CO}{}_{2}\vec{\leftarrow }\mathrm{CO}+O$$

A further reaction (see Figure 4, intermediate **f** to **k**) is shown in Equation 10, whereby an adsorption site is exchanged for a hydrogen atom. If both adsorption sites in **f** were identical, it would be difficult to understand why an oxygen atom would remain bound, especially when the large number of active hydrogen atoms present under the reaction conditions is considered. If, on the other hand, a step exists on the metal surface or an impurity atom (a ZnO site for example) is next to a Cu adsorption site, the likelihood of reaction (10) in Figure 4 is easier to understand, because the two adsorption sites in **f** are no longer identical.

(10) $$\mathrm{HCO}{}_{2}\vec{\leftarrow }\mathrm{HCO}\left(\mathrm{OH}\right)$$

An analogous consideration holds for the form of the reacting hydrogen. It can be transferred as an H atom (radical), as a hydride (on a metal), or as a proton (on an oxo group) plus an electron. In each case, the participation of the catalyst is necessary, as it, at the very least, exchanges charge with the adsorbates. These processes must also be investigated in the context of a microkinetic description to clarify, for example, whether some of the processes are simultaneously active. Illustrating the fine differences in the reactivity of metal–hydrogen species, studies^[144]^ have shown that CO_2_ can be activated by insertion into the metal−H bond of a dihydrido complex to form formate. The local electronic structure is decisive for this process; an analogous monohydrido complex does not show the same reactivity, but is electronically indistinguishable from the dihydrido analogue in NMR spectra. Both Zn and Cu are able to form hydrides as a crystalline substance if they come from a precursor compound of low valency (M_2_O) and are converted with atomic hydrogen. This situation occurs during electroreduction. Such hydrides cannot, however, be produced though synthesis from the elements. It is not known whether hydrides are formed during the reduction of Cu_*x*_O under a high hydrogen pressure. In this context it is interesting to note that the polymeric substance “CuH” exhibits the same red‐brown coloration as Cu_2_O and even contains embedded residual oxygen. Thus, the idea that electron‐poor copper is important for the reduction of CO_2_ benefits from a new aspect resulting from the so far largely overlooked possibility of CuH formation (along with its hydride adduct CuH_4_^3−^) as the active form of the copper MeOH catalyst.

The reactions in Figure 4 can be assumed to be highly sensitive to the local electronic structures and the local morphological conditions.^[83]^ These two properties are, even with the analytical methods used today, hardly distinguishable from each other when based on a matrix of Cu metal and a carrier phase. A surface‐sensitive structural analysis under the reaction conditions^[110b]^ is needed which does not influence or change a termination layer that dynamically responds to its surrounding chemical potential. If the system is extracted from the reaction environment, an unambiguous surface state^[47h]^ can indeed be found. However, this state is not necessarily the most reactive one.^[136]^ Thus, it becomes evident that a rigorous description of a functioning catalyst is, even today, still a problem without a solution in sight.^[145]^ That the current state of research can be confusing and seemingly contradictory to the outside observer is due to the fact that many studies are carried out with different systems that are not properly distinguished (although they may all be active). Furthermore, these studies are not always critical enough with respect to the methodological sensitivities[^88b^, ^113b^, ^146^] to unambiguously detect a structure present as a fraction of a monolayer which may not exist under standard conditions. Many conclusions in the literature lose their contradictory character if the boundary conditions, which are often not discussed when making a very explicit claim, are considered.

Under mild reaction conditions, different surface structures co‐exist and will, therefore, generate a number of reaction products from CO_2_. For example, this happens in electroreduction, with its low thermal excitation for restructuring at the electrode. In situ XAS measurements on the deposition of Cu on a gold electrode showed[^97a^, ^116^] that, during the process, mixtures of Cu^+^ and some Cu^0^ arise from the disproportionation of Cu^+^ into Cu^2+^ and Cu^0^ instead of only metallic copper, which makes up the main phase. If this mixture is used under electrochemical reduction conditions for CO_2_ in an alkaline electrolyte and a strong negative potential, the resulting material may not only be copper, but rather a metal modified with oxygen (OH).[^102a^, ^147^] This material may, therefore, have different catalytic characteristics than pure copper. It is also plausible that the selectivity[^47e^, ^97a^] of copper can be controlled using a deliberately chosen pretreatment with oxygen. A switching between formate and CO generation can be observed depending on the pH of the electrolyte and thus the surface concentration of H_3_O^+^.^[148]^ The decisive value is the local pH value, which can deviate significantly from the pH of the electrolyte as a result of diffusion processes at the electrode.^[8a]^ The local pH value affects the degree to which Cu oxides—the thermodynamically stable forms of the electrode in the non‐acid environment—are generated. The concept of a partially inhibited (“frustrated”) phase change^[98]^ between the metal and oxide brought on by an applied reductive potential likely describes well the state of active electrocatalysts in the hydrogenation of CO_2_.

Under harsh conditions, on the other hand, only states such as “alloy,” “surface oxide,” or “perimeter oxide” will exist and, therefore, only a small number of products will be generated. An example of this is the high‐pressure synthesis of methanol. If, however, stable co‐catalysts such as transition‐metal atoms and their low valency oxo compounds are present,[^66d^, ^66e^] higher alcohols can, in addition to methanol, also be generated in relevant quantities under extreme conditions. This result makes it clear that the reaction network shown in Figure 4 can be realized in many different and parallel pathways with reactive copper. The monotony of the highly selective methanol synthesis is determined by the singular synergy of copper and zinc leading to the uniformly modified copper metal “methanol copper.” The synergy is a consequence of the long process of material optimization of the catalyst and not a distinctive characteristic of the copper‐CO_2_‐H_2_ system.

## 12 Epilogue

The reduction of CO_2_ leads to a wide array of products. As fuels, some of the products will play a strategic role in future energy regimes along with a circular economy and the storage of fluctuating renewable electricity. The products make possible a concept such as the chemical battery, with which nearly an unlimited supply of renewable energy can be stored and transported. Only thanks to the chemical battery concept will a global trade of renewable energy be possible and replace the trade of fossil energy carriers. Furthermore, the chemical battery enables the use of renewable energy in the mobility sector, where, most notably, high‐performance applications with electric batteries are difficult to implement. The similarities of the physicochemical material characteristics of chemical batteries and fossil fuels allow the further use of contemporary converters (motors and turbines). The chemical battery “hydrogen” is only able to fill this role in a limited way and in addition requires new infrastructure for transport to the user.

Other products resulting from the hydrogenation of CO_2_ will change parts of the resource infrastructure of the chemical industry. In some cases, the use of CO_2_ as a building block for chemical synthesis will lead to new methods for the production of complex chemical products. Such processes will contribute to a reduction of CO_2_ emission from the chemical industry by simplifying synthetic routes and thus saving fossil raw materials and process energy. All of these applications are based on the chemistry of CO_2_ in different reaction environments, which, at a fundamental level, is well‐understood.

The objective of the hydrogenation of CO_2_ is to use it as an energy carrier in the carbon cycle economy. Its use as a scavenger for fossil emissions would be way too energy‐intensive and costly and could thus not contribute to the mass‐reduction problem of reducing emissions (Gt per annum). The prime objective must be to stop the emission of CO_2_ from fossil sources, and this can only happen if we replace fossil by renewable energy carriers on a global scale. This in turn requires the concept of chemical batteries as a basis and with it the application of well‐understood catalysis technologies.

Scientific research into CO_2_ chemistry has devoted much attention to a small number of molecules such as methanol and olefins. A wide range of other possible sophisticated reactions has received much less focus in research. This choice can be justified by the challenge of both the urgency and the scale of the processes based on CO_2_ as a raw material. In this field, as well as with the production and purification of CO_2_ (from air, for example), there are a multitude of scientific problems still to be solved. At the same time, though, many synthetic and catalytic studies in CO_2_ chemistry do not identify any pathways to scalable processes for the next two decades. One suggestion to overcome this is that, in the future, authors should either realistically evaluate their prospects for contributing to climate protection and reflect on these considerations during their experiments, or the generation of knowledge should be expressed as the motivation of the work.

This applies also to the literature on methanol synthesis and higher alcohols reviewed here. Comprehensive and highly detailed studies with seemingly contradictory conclusions describe a reaction network for the hydrogenation of CO_2_, which is, depending on the conditions and catalyst, branched in different ways. The multiple chemical states of the central element copper have only now begun to be recognized with respect to the molecular geometry and the presence of modifying atoms such as oxygen and structural dynamics. Some controversies in the literature have arisen from the interpretation of experimental results that have been reported without adequate critical reflection on the rich and dynamic structural details of the copper. The methods were not surface‐sensitive enough, did not contain enough evidence to support the conclusions, or did not sufficiently consider gaps in chemical potential connected with the dynamic reaction of an active catalyst. These insights have been possible thanks to many challenging operando experiments. Theory has also been responsible for supplying critical motivation for the atomic‐level understanding of reaction pathways and the structural peculiarities that lie at their core. Nevertheless, today, a comprehensive description of the fundamental aspects of a reacting system consisting of a catalyst, carrier, and the reaction conditions is still not available.

However, the analysis provided here concludes that the reduction of CO_2_ to methanol proceeds by the formate/RWGS route (see Figure 4) at bifunctional centers which are likely situated along the perimeter line between copper metal and partially reduced ZnO. Oxalate can also disproportionate on this line as dimerized active CO_2_ and produce HCO which, at metallic centers, can then produce either methanol or lead to higher alcohols depending on the local electronic structure. To differentiate the pathway taken, the chemical potential of the active hydrogen is likely decisive. The potential can, particularly in electrochemical reduction, be varied over a wide range by means of an applied voltage and the pH value of the electrolyte. In this way, a number of higher hydrocarbons can be produced which cannot be formed in gas‐phase chemistry using the Cu/ZnO system.

The assumption cannot be substantiated here^[81]^ that contemporary methods for the industrial hydrogenation of CO_2_ to methanol are not of adequate stability. It is correct that the productivity of modern systems is only technically satisfactory if the feed gases are recycled, something that places particular demands on controlling the impurity levels of gases and incurs cost to enable recirculation under pressure. However, there has been no description of a system in the academic literature that has even a chance at technical realization when employing less or no recirculation. It seems, then, that this challenge must be met by a fundamentally new approach, beginning, perhaps, with CO as the input gas (which can be readily produced from CO_2_) to produce a mixture of methanol and higher alcohols. An opportune use of the resulting products could be fuels[^49f^, ^53a^] to avoid excessive separation and cracking efforts of the raw mixtures. The CO_2_ hydrogenation chemistry may then approach the well‐known FT chemistry.

If, as is the case here, the fundamentals of a complex reaction sequence in a network of processes are unclear, an effective recourse is the employment of model systems (copper single crystals, for example). Complexity can be reduced in this way to gather detailed information[^145^, ^149^] on elementary processes and their dependence on the structure of the model catalyst. This strategy has provided much critical insight into the mechanisms of CO_2_ reduction also used here for discussion. The intended reduction of complexity excludes the detection of significant aspects of the reactivity under high‐performance conditions that depend on the chemical constitution and dynamics of the metal surface of the working catalyst. This shortcoming results in the well‐known “science gaps” in catalysis research. To overcome this challenge, the application of artificial intelligence and its diverse methods may result in decisive contributions leading to structure–function correlations that can bridge the gaps.

A major impediment in this respect is the incomplete and poorly structured presentation of comprehensive and high‐quality results. Attempts at collecting all the descriptions of CO_2_ reduction at interfaces using modern methods of digital catalysis research would quickly fail for this reason. Thus, one point of urgency in the current Review is to declare that a standardization of methods and descriptions in future theoretical and experimental studies on the hydrogenation of CO_2_ should be introduced as a precedent for the execution and documentation of future research on catalytic processes. The result would be a “handbook” defining standards and general research guidelines for synthesis, testing, and functional characterization as well as documentation in the framework of a certified metrology. In this way, a minimal standard would be established for future work without restricting the creative developments of tomorrow's research. All research carried out according to these norms would offer a fount of data^[150]^ on which robust (complex) structure–function relationships and extrapolations in the space of materials and the corresponding reaction conditions would be possible. The importance of the hydrogenation of CO_2_ for our future justifies this undertaking without hesitation.

## Conflict of interest

The authors declare no conflict of interest.

## Biographical Information

*Robert Schlögl (born in Munich in 1954) has been director at the Fritz Haber Institute of the Max Planck Society in Berlin since 1994 and founding director of the MPI for Chemical Energy Conversion in Mülheim a.d. Ruhr since 2011. In 2020, he was elected Vice President of the National Academy Leopoldina. He is Honorary Professor at the TU Berlin, Humboldt University of Berlin, University of Duisburg‐Essen, and Ruhr University of Bochum as well as member of acatech and BBAW. His research focuses on heterogeneous catalysts, with the aim to combine scientific with technical applicability, as well as nanochemically optimized materials for energy storage*.